# Comprehensive transcriptome analysis of different potato cultivars provides insight into early blight disease caused by *Alternaria solani*

**DOI:** 10.1186/s12870-023-04135-9

**Published:** 2023-03-08

**Authors:** Radha Sivarajan Sajeevan, Ingi Abdelmeguid, Ganapathi Varma Saripella, Marit Lenman, Erik Alexandersson

**Affiliations:** 1 Department of Plant Protection Biology, Swedish University of Agricultural Sciences, 23422 Lomma, Sweden; 2 Department of Botany and Microbiology, Faculty of Science, Helwan University, Cairo, EG-11795 Egypt; 3 Department of Plant Breeding, Swedish University of Agricultural Sciences, 23422 Lomma, Sweden; 4 CropTailor AB, Department of Chemistry, Division of Pure and Applied Biochemistry, Lund University, Lund, Sweden

**Keywords:** Potato, Early blight, *Alternaria solani*, Transcriptome, Transcription factors, Jasmonic acid, Ethylene, Photosynthesis, Starch metabolism, Terpenes

## Abstract

**Background:**

Early blight, caused by the necrotrophic fungal pathogen *Alternaria solani*, is an economically important disease affecting the tuber yield worldwide. The disease is mainly controlled by chemical plant protection agents. However, over-using these chemicals can lead to the evolution of resistant *A. solani* strains and is environmentally hazardous. Identifying genetic disease resistance factors is crucial for the sustainable management of early blight but little effort has been diverted in this direction. Therefore, we carried out transcriptome sequencing of the *A. solani* interaction with different potato cultivars with varying levels of early blight resistance to identify key host genes and pathways in a cultivar-specific manner.

**Results:**

In this study, we have captured transcriptomes from three different potato cultivars with varying susceptibility to *A. solani*,  namely Magnum Bonum, Désirée, and Kuras, at 18 and 36 h post-infection. We identified many differentially expressed genes (DEGs) between these cultivars, and the number of DEGs increased with susceptibility and infection time. There were 649 transcripts commonly expressed between the potato cultivars and time points, of which 627 and 22 were up- and down-regulated, respectively. Interestingly, overall the up-regulated DEGs were twice in number as compared to down-regulated ones in all the potato cultivars and time points, except Kuras at 36 h post-inoculation. In general, transcription factor families WRKY, ERF, bHLH, MYB, and C2H2 were highly enriched DEGs, of which a significant number were up-regulated. The majority of the key transcripts involved in the jasmonic acid and ethylene biosynthesis pathways were highly up-regulated. Many transcripts involved in the mevalonate (MVA) pathway, isoprenyl-PP, and terpene biosynthesis were also up-regulated across the potato cultivars and time points. Compared to Magnum Bonum and Désirée, multiple components of the photosynthesis machinery, starch biosynthesis and degradation pathway were down-regulated in the most susceptible potato cultivar, Kuras.

**Conclusions:**

Transcriptome sequencing identified many differentially expressed genes and pathways, thereby contributing to the improved understanding of the interaction between the potato host and *A. solani*. The transcription factors identified are attractive targets for genetic modification to improve potato resistance against early blight. The results provide important insights into the molecular events at the early stages of disease development, help to shorten the knowledge gap, and support potato breeding programs for improved early blight disease resistance.

**Supplementary Information:**

The online version contains supplementary material available at 10.1186/s12870-023-04135-9.

## Background

Potato (*Solanum tuberosum*) is the third most important food crop after wheat and rice worldwide [1, 2]. Apart from being a food source, potato starch is widely used as a raw material for various industrial purposes. Potato cultivation faces many biotic threats, of which early blight is one of the most serious diseases. It is caused by a range of necrotrophic fungi belonging to *Alternaria* spp., with *A. solani* being the most aggressive to potato [3]. In Sweden, *A. solani* is considered to be the main causal agent for early blight in potatoes [4]. The infection starts in the older leaves as small dark spots; under favorable climatic conditions, these will enlarge to form large necrotic lesions and subsequent defoliation. The defoliation will result in reduced yield. Currently, early blight is controlled by the repeated application of fungicides [5], and if left uncontrolled, the tuber yield loss can reach up to 40 to 50% [6, 7]. The fungicides in use are becoming less potent due to mutations in the active sites and fungicide-resistant populations of *A. solani*, which have been reported in multiple countries [8–12]. In addition, excess or prolonged application of fungicides may lead to the accumulation of these chemicals in the soil and water sources, which may become environmental contamination and food safety problems [13–15].

Disease prevention based on resistant cultivars is the best long-term solution in potatoes for effectively managing early blight [15]. Even if no complete resistance has been identified, it is well-known that potato cultivars have varying resistance levels against early blight. A few studies have been conducted to identify early blight resistance genes and unravel the plant responses with little success [16–18]. The resistance is suggested to be polygenic and thus quantitative in nature. It is also linked to the foliage maturity of potato cultivars [15, 19, 20]. It has been shown that late-maturing potato cultivars are more resistant to early blight and vice versa [15–17, 20, 21]. Quantitative trait loci (QTLs) for foliar and tuber early blight resistance were identified on chromosomes 1, 5, 6, 7, 11, and 12, and chromosomes 1, 2, 3, 4, 8, 11, and 12, respectively, based on a tetraploid potato segregating population [17]. The QTLs identified in chromosomes 5 and 11 were independently mapped for foliage maturity (leaf defoliation). Another study by Zhang [16] identified five different QTLs on chromosomes 4, 5, 9, 11, and 12 for foliar resistance in a diploid segregating population, of which QTLs in chromosomes 4 and 5 overlapped with foliar maturity. More recently, in field trials, foliage resistance against early blight was tested for two consecutive years (2018 and 2019) using 271 progenies obtained from a cross between B0692-4, a resistant clone, and the susceptible cultivar Harley Backwell. This study identified three and six QTLs against early blight resistance for the years 2018 and 2019, respectively. Two QTLs that mapped on chromosome 5 were common for both years and overlapped with foliage maturity. In 2018, one minor QTL was mapped to chromosome 7; in 2019, four minor QTLs were mapped on chromosomes 2, 3, 8, and 12, unrelated to foliage maturity [15]. However, many of these studies had limitations due to small population sizes or incomplete linkage maps.

A limited number of transcriptome-based studies have been conducted to understand the molecular changes occurring during *A. solani* leaf infection. Microarrays were used to study the early blight susceptible potato cultivar Désirée, as well as salicylic acid (SA) deficient and jasmonic acid (JA) insensitive lines at 24, 72, and 120 h post *A. solani* inoculation. The study shows a high number of differentially expressed genes (DEGs) in the SA deficient line compared to wild type and JA insensitive line [22]. In order to capture and understand the early and late plant responses to *A. solani* infection, Brouwer et al. [18] carried out RNA sequencing from *A. solani* inoculated potato leaves of the susceptible cultivar Désirée. The transcriptome was developed starting from an early time point at 1 h after inoculation (the initial stage of conidia germination), followed by 6, 12, 24, and 48 h post-infection (hpi), and studied the changes in expression of potato as well as *A. solani* transcripts. The up-regulated transcripts were mainly linked to biotic stress tolerance and pathogen defense. Currently, these are the only transcriptome studies to understand the potato—*A. solani* interaction. Here we present gene expression profiling from different potato cultivars with varying levels of resistance to early blight to understand the genotype-specific molecular responses better. With this objective, we analyzed the transcriptomes at two different time points after *A. solani* inoculation from three potato cultivars with varying levels of early blight resistance. Changes in the transcriptomes generated by RNA sequencing identified key pathways and genes as well as potential molecular mechanisms during infection that showed a clear cultivar-specific response.

## Results

### Disease severity analysis in different potato cultivars

**Fig. 1:**
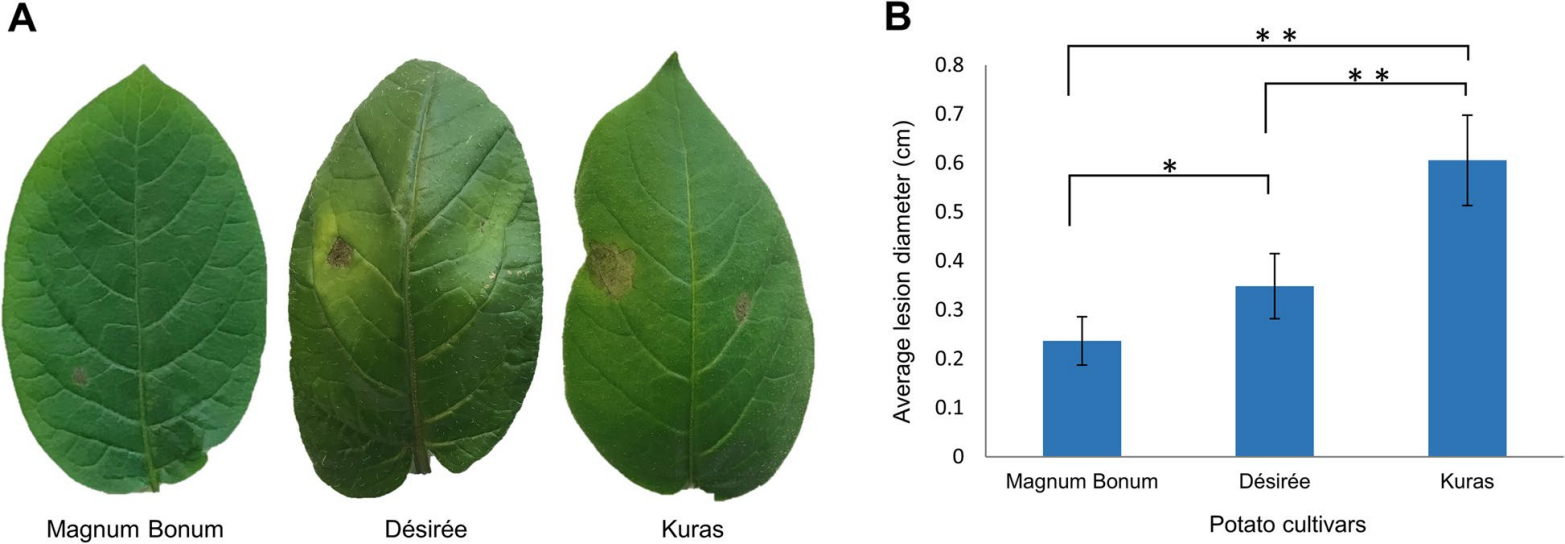
Disease severity of different potato cultivars to early blight disease at 96 hpi. **A** representative photographs show the phenotype of necrotic lesion, **B** average lesion diameter*.* Graph showing mean values ± SE. Significant differences were determined by ANOVA using Tukey’s HSD analysis (**p value < 0.01 and *p value < 0.05)

As previously reported [6, 11, 17, 22], a screening of different potato cultivars showed that Magnum Bonum, Désirée and Kuras differed in their susceptibility to *A. solani*. Potato leaves inoculated with *A. solani* spores started showing visual symptoms (necrosis) after 24 h while the control plants inoculated with sterile water were intact. The infection efficiency was more than 90% (data not shown) and there was a significant difference in necrotic lesion diameter between the three potato cultivars (Fig. 1). The partially susceptible potato cultivar Kuras showed the largest necrotic lesion of 0.61 cm followed by 0.35 cm for Désirée compared to 0.24 cm for the partially resistant cultivar Magnum Bonum (Fig. 1).

### Transcriptome sequencing, mapping, and principal component analysis

To study the changes in gene expression, we sequenced the total RNA from the three different potato cultivars at 18 and 36 h post *A. solani* inoculation (before and after necrotic spots appeared in the susceptible cultivar Kuras). Overall, a total of 128.82 and 131.52 million reads were generated from control and *A. solani* inoculated leaf samples, respectively. The reads with adaptor contamination and low base quality were removed. An average of 89.13 and 87.63% of control and *A. solani* inoculated sequence reads were mapped to the potato genome (DM 1–3 516 R44 v6.1), respectively. The overall statistics are given in Table 1. The principal component analysis (PCA) was carried out to visualize how each of the samples were clustering based on the variation in gene expression. There was a clear difference between the control and inoculated samples, forming four clear, discrete groupings. Irrespective of the potato cultivars, the samples were primarily divided based on control and *A. solani* inoculation (PC1), 18 and 36 h time points (PC2). The PC1 and PC2 indicate the first and second largest sources of variation within the dataset. PC1 representing the highest variance (50%) dividing the control and *A. solani* inoculated samples, was substantially higher than PC2 representing 15% of the variance dividing the 18 and 36 h time points. The analysis showed that all three biological replicates for each time point and potato cultivars clustered closely (Additional Fig S1).

**Table 1:** RNA sequencing statistics of different *S. tuberosum* during *A. solani* infection. Total reads (million), percentage of reads mapping to potato reference genome per time point. The data shown are the average of three biological replicates

	**Potato cultivars and Time Point (Hours Post Inoculation)**							
	**Magnum Bonum**			**Désirée**			**Kuras**	
	**18**	**36**	**18**		**36**	**18**		**36**
Total reads for control (Million)	26.10	21.40	18.77		22.17	20.43		19.93
Total reads for *A. solani* inoculated (Million)	25.27	21.57	23.70		18.73	20.13		22.13
Control sequence reads uniquely mapped to *S. tuberosum* reference genome (%)	89.17	88.80	89.89		89.49	87.89		89.52
*A. solani* inoculated sequence reads uniquely mapped to *S. tuberosum* reference genome (%)	90.48	86.33	88.92		87.57	86.42		86.38

### Differential gene expression in response to *A. solani* infection, a global view

**Fig. 2:**
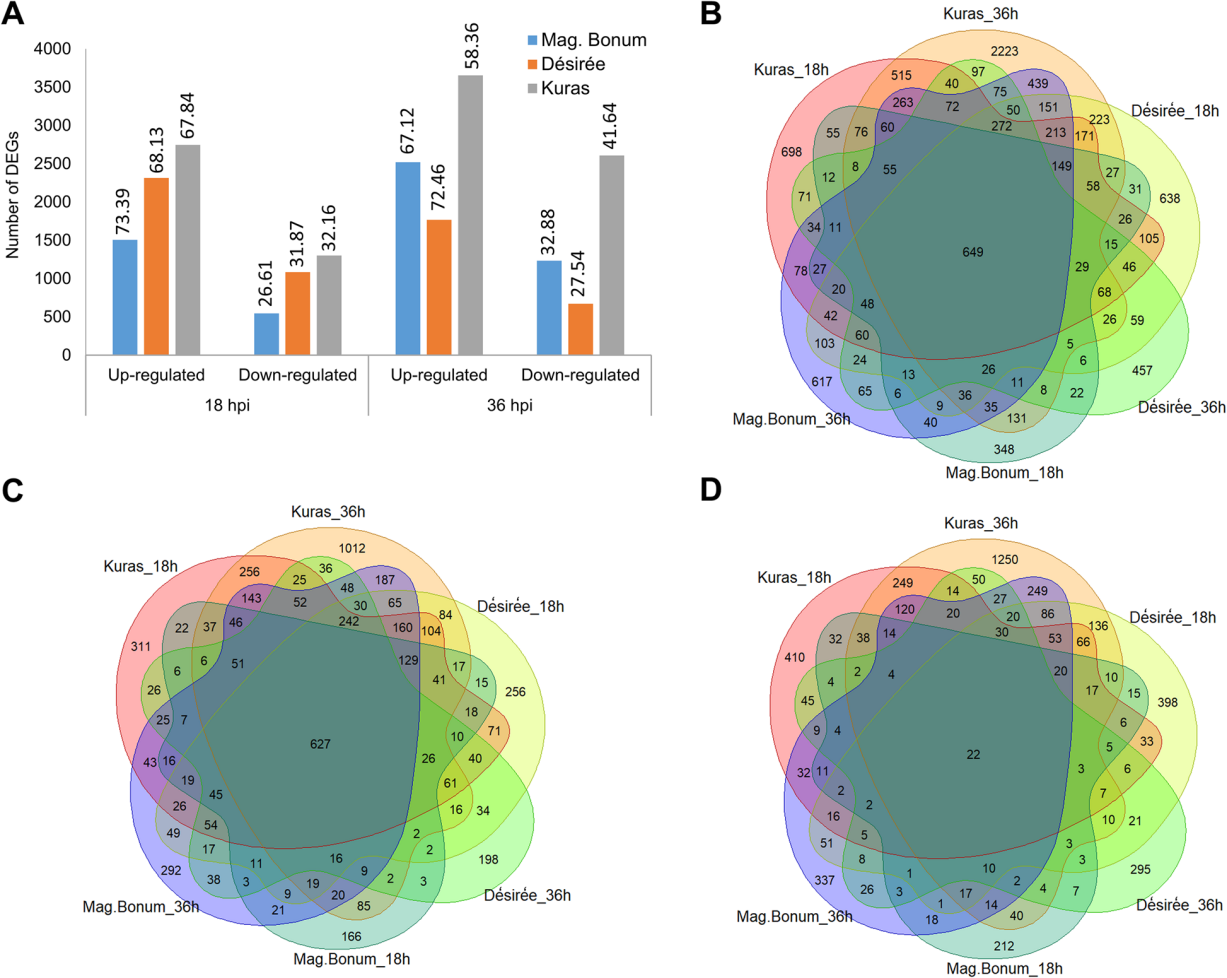
Number of DEGs identified from different potato cultivars and time points. **A** Up- and down-regulated DEGs identified from Magnum Bonum (Mag. Bonum), Désirée, and Kuras at 18 and 36 hpi, **B** Venn diagram showing the overlap between the total DEGs of Magnum Bonum (Mag. Bonum), Désirée, and Kuras at 18 and 36 hpi, **C** Venn diagram showing the overlap between the up-regulated DEGs of Magnum Bonum (Mag. Bonum), Désirée, and Kuras at 18 and 36 hpi, respectively, **D** Venn diagram showing the overlap between the down-regulated DEGs of Magnum Bonum (Mag. Bonum), Désirée, and Kuras at 18 and 36 hpi, respectively

The differential gene expression analysis was carried out to understand the global changes in the transcripts when different potato cultivars were inoculated with *A. solani*. Significant differences in gene expression were noticed between the potato cultivars and at the two-time points studied. Magnum Bonum, a partially resistant early blight disease cultivar, had the least number of DEGs at 18 hpi (2052), followed by Désirée (3398), whereas the maximum numbers of DEGs were identified for the partially susceptible cultivar, Kuras (4046). At 36 hpi, the numbers were increased in Magnum Bonum (3753) and in Kuras (6261) but decreased in Désirée (2440) (Fig. 2A). Of the DEGs identified, interestingly, the number of up-regulated transcripts was significantly higher than the down-regulated ones in all the potato cultivars at 18 and 36 hpi. Many of the top 10 up-regulated DEGs were the same for Magnum Bonum and Kuras, but there was a higher Log2 fold expression in Kuras. A few DEGs Soltu.DM.01G048780.1, Soltu.DM.09G024040.1, and Soltu.DM.01G040940.1 identified at 36 hpi in Magnum Bonum were identified at 18 hpi from Kuras with a higher Log2 fold expression. In fact, among all the 18 and 36 hpi top 10 up-regulated Magnum Bonum transcripts, only one (Soltu.DM.12G027800.1) did not show some degree of up-regulation in Kuras or Désirée at the same time point.

**Fig. 3:**
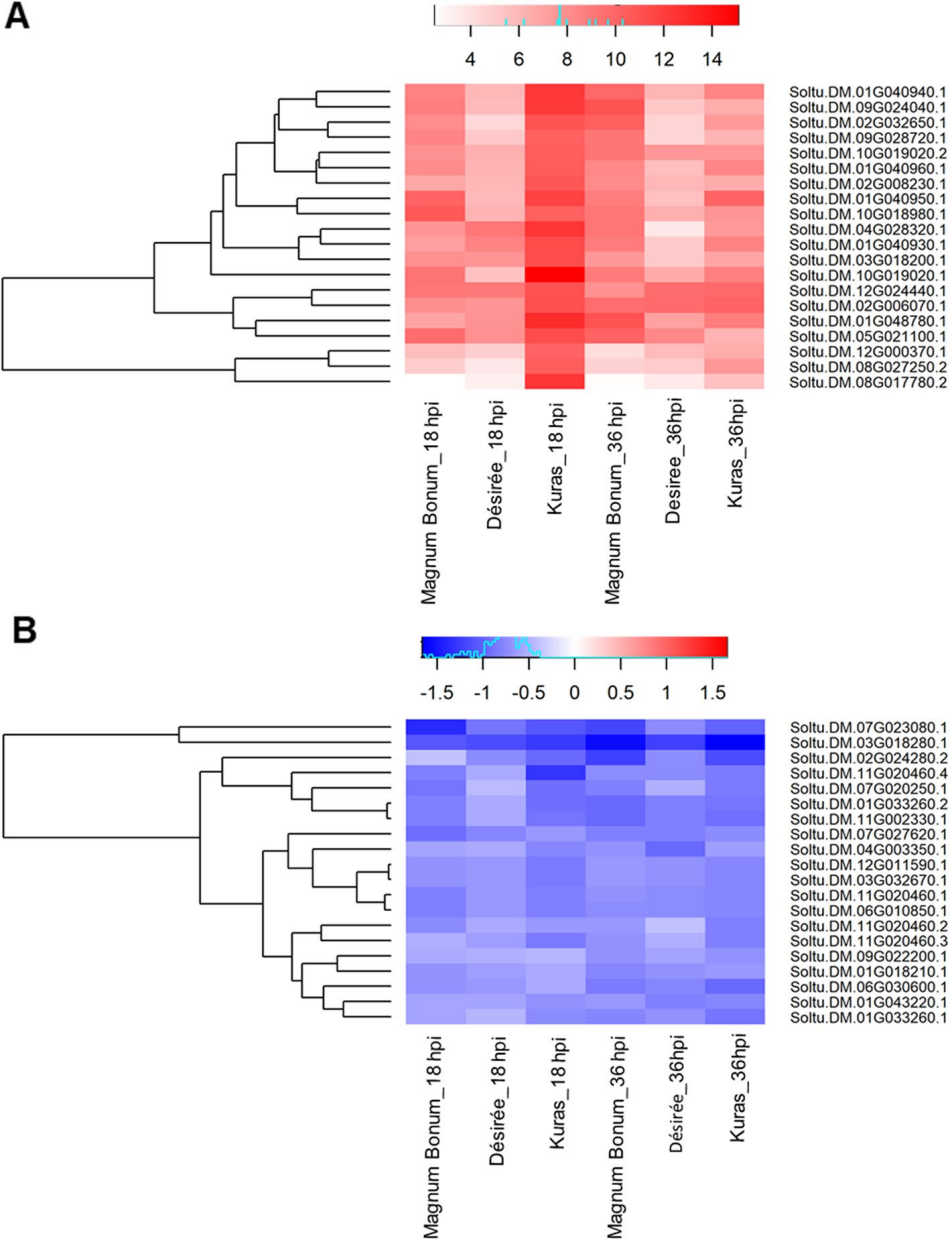
Heat map showing the **A** top 20 up- and **B** down-regulated common DEGs from different potato cultivars at 18 and 36 hpi

In contrast to the most up-regulated DEGs, only one overlap between Magnum Bonum and Kuras was observed among the top down-regulated DEGs. In fact, only two out of all the 18 and 36 hpi top 10 down-regulated Magnum Bonum transcripts showed some degree of down-regulation in either Kuras or Désirée at the same time point. The remaining eight transcripts at 18 hpi that were specific for Magnum Bonum included a glutamate-1-semialdehyde 2,1-aminomutase (Soltu.DM.04G005660.1), an abscisic acid responsive elements-binding factor (Soltu.DM.07G000240.4) and a pleckstrin homology (PH) domain-containing protein. The top 10 DEGs that are up- and down-regulated are provided in Table 2. The complete list of DEGs detected can be found in Additional files 1,2,3. To identify the common and unique DEGs for different potato cultivars and time points, Venn diagrams were generated (Fig. 2B-D). A total of 994 DEGs were common at 18 hpi of which 915 were up-regulated, and 77 were down-regulated. Similarly, at 36 hpi, 1210 transcripts were found to be common, of which 1075 and 135 were up- and down-regulated, respectively (Additional Fig. S2A-F). We found 649 genes commonly expressed between the time points and different potato cultivars analyzed, of which, interestingly, 627 were up-regulated and 22 were down-regulated (Fig. 2B-D). Even though these are commonly expressed transcripts, there was a significant difference in the fold change values between the potato cultivars and time points. A significant up-regulation was observed for a peroxidase superfamily protein (Soltu.DM.10G019020.1) and allene oxide synthase (Soltu.DM.01G048780.1) in Kuras 18 hpi and fructose-bisphosphate aldolase (Soltu.DM.02G024280.2) was significantly down-regulated across the potato cultivars and time points analyzed. The top 20 up- and down-regulated common transcripts were displayed in a heat map (Fig. 3A&B). The complete details of the transcripts ID, gene function, corresponding PGSC_DM_V403 gene ID, PGSC functional gene annotation, and the corresponding Log2 fold change were provided in Additional file 4.

**Table 2:** Top 10 up- and down-regulated DEGs in Magnum Bonum, Désirée, and Kuras potato cultivars upon infection with *A. solani* at 18 and 36 hpi. Gene name. gene description. Log2 Fold change. and the adjusted P-value (Padj) of infected compared to control are shown

Gene name	Description	Log2 fold change
**18 hpi**	**Magnum Bonum**	**Up-regulated**
Soltu.DM.10G018980.1	Peroxidase superfamily protein	10.68
Soltu.DM.08G011070.1	Ankyrin repeat family protein	10.67
Soltu.DM.01G040950.1	terpene synthase	10.14
Soltu.DM.05G021100.1	Rhamnogalacturonate lyase family protein	9.69
Soltu.DM.07G013650.1	cytochrome P450, family 716. subfamily A, polypeptide	9.68
Soltu.DM.12G027800.1	KNOTTED1-like homeobox gene	9.59
Soltu.DM.06G016360.1	terpene synthase	9.44
Soltu.DM.09G027720.3	MLP-like protein	9.41
Soltu.DM.10G019020.1	Peroxidase superfamily protein	9.33
Soltu.DM.01G035900.1	zinc induced facilitator-like	9.22
**18 hpi**	**Magnum Bonum**	**Down-regulated**
Soltu.DM.04G005660.1	glutamate-1-semialdehyde-2.1-aminomutase	-9.91
Soltu.DM.04G020260.3	chromatin remodeling	-8.66
Soltu.DM.08G021790.1	lectin protein kinase family protein	-8.64
Soltu.DM.11G016910.2	abscisic acid responsive elements-binding factor	-8.42
Soltu.DM.07G000240.4	response regulator	-8.38
Soltu.DM.12G029710.4	origin recognition complex subunit	-8.28
Soltu.DM.08G023390.2	pleckstrin homology (PH) domain-containing protein	-8.09
Soltu.DM.06G024580.1	respiratory burst oxidase homologue D	-8.02
Soltu.DM.02G029210.2	SAP domain-containing protein	-7.89
Soltu.DM.03G021350.1	Calcium-binding endonuclease/exonuclease/phosphatase family	-7.83
**36 hpi**	**Magnum Bonum**	**Up-regulated**
Soltu.DM.09G024040.1	carboxyesterase	10.97
Soltu.DM.01G048780.1	allene oxide synthase	10.95
Soltu.DM.02G032650.1	Peroxidase superfamily protein	10.31
Soltu.DM.05G021100.1	Rhamnogalacturonate lyase family protein	10.15
Soltu.DM.04G020660.1	cytochrome P450, family 71, subfamily B, polypeptide	10.13
Soltu.DM.01G040940.1	terpene synthase	9.95
Soltu.DM.02G006070.1	BURP domain-containing protein	9.68
Soltu.DM.09G026810.1	Protein kinase superfamily protein	9.60
Soltu.DM.06G018620.1	serine-type endopeptidase inhibitors	9.51
Soltu.DM.07G003530.1	copper ion binding;electron carriers	9.50
**36 hpi**	**Magnum Bonum**	**Down-regulated**
Soltu.DM.09G026790.2	S-adenosyl-L-methionine-dependent methyltransferases superfamily protein	-9.13
Soltu.DM.07G016780.3	ethylene-forming enzyme	-8.93
Soltu.DM.10G004560.2	diacylglycerol kinase	-8.73
Soltu.DM.08G021790.1	lectin protein kinase family protein	-8.68
Soltu.DM.12G029610.2	protochlorophyllide oxidoreductase B	-8.53
Soltu.DM.01G043110.1	Phosphatidylinositol-4-phosphate 5-kinase family protein	-8.43
Soltu.DM.05G009760.8	root hair specific	-8.13
Soltu.DM.04G034990.3	J-domain protein required for chloroplast accumulation response	-8.07
Soltu.DM.06G033730.2	Helicase/SANT-associated. DNA binding protein	-8.03
Soltu.DM.09G020140.2	methylcrotonyl-CoA carboxylase alpha chain. mitochondrial / 3-methylcrotonyl-CoA carboxylase 1 (MCCA)	-8.01
**18 hpi**	**Désirée**	**Up-regulated**
Soltu.DM.02G018060.1	Protein of unknown function (DUF_B2219) domain containing protein	10.13
Soltu.DM.01G036130.3	Transducin/WD40 repeat-like superfamily protein	10.12
Soltu.DM.10G000710.3	Protein kinase superfamily protein	9.24
Soltu.DM.04G028320.1	laccase	9.18
Soltu.DM.12G024440.1	Lactoylglutathione lyase / glyoxalase I family protein	9.10
Soltu.DM.06G016360.1	terpene synthase	9.04
Soltu.DM.10G005990.2	Protein BPS1, chloroplastic	8.77
Soltu.DM.08G024320.1	Protein of unknown function (DUF1639)	8.74
Soltu.DM.07G027860.3	amino acid permease	8.73
Soltu.DM.07G027440.1	emp24/gp25L/p24 family/GOLD family protein	8.67
**18 hpi**	**Désirée**	**Down-regulated**
Soltu.DM.01G043130.2	ankyrin repeat-containing 2B	-9.90
Soltu.DM.07G016780.3	ethylene-forming enzyme	-9.80
Soltu.DM.04G004430.2	histone acetyltransferase of the CBP family	-8.95
Soltu.DM.11G022970.4	auxin response factor	-8.75
Soltu.DM.01G034500.2	conserved hypothetical protein	-8.72
Soltu.DM.04G000750.3	Disease resistance protein (CC-NBS-LRR class) family	-8.53
Soltu.DM.06G020110.3	conserved hypothetical protein	-8.49
Soltu.DM.02G005200.3	Calcium-binding EF hand family protein	-8.39
Soltu.DM.02G005200.2	Calcium-binding EF hand family protein	-8.38
Soltu.DM.10G014560.2	TIP41-like family protein	-8.38
**36 hpi**	**Désirée**	**Up-regulated**
Soltu.DM.08G005960.3	O-acetylserine (thiol) lyase isoform C	10.82
Soltu.DM.02G006070.1	BURP domain-containing protein	9.74
Soltu.DM.12G024440.1	Lactoylglutathione lyase / glyoxalase I family protein	9.66
Soltu.DM.04G024810.3	UDP-sugar pyrophosphorylase	9.54
Soltu.DM.01G002240.1	2-oxoglutarate (2OG) and Fe(II)-dependent oxygenase superfamily protein	9.49
Soltu.DM.12G021490.1	cellulose synthase like G3	9.40
Soltu.DM.12G026250.2	photosystem I light harvesting complex gene	9.03
Soltu.DM.09G018860.2	multidrug resistance-associated protein	8.98
Soltu.DM.01G047660.1	ARP protein (REF)	8.42
Soltu.DM.05G021100.1	Rhamnogalacturonate lyase family protein	8.37
**36 hpi**	**Désirée**	**Down-regulated**
Soltu.DM.07G016780.3	ethylene-forming enzyme	-9.98
Soltu.DM.12G023130.1	Zinc finger C- × 8-C- × 5-C- × 3-H type family protein	-8.79
Soltu.DM.10G029600.1	UDP-Glycosyltransferase superfamily protein	-8.76
Soltu.DM.05G009830.1	cyclin-related	-8.76
Soltu.DM.03G007180.4	DUF4336 domain containing protein	-8.54
Soltu.DM.01G006350.2	BRI1 suppressor 1 (BSU1)-like	-8.48
Soltu.DM.01G043130.2	ankyrin repeat-containing 2B	-8.38
Soltu.DM.09G013230.4	splicing factor PWI domain-containing protein	-8.14
Soltu.DM.05G019260.1	DHHC-type zinc finger family protein	-8.09
Soltu.DM.05G009830.5	cyclin-related	-8.03
**18 hpi**	**Kuras**	**Up-regulated**
Soltu.DM.10G019020.1	Peroxidase superfamily protein	15.09
Soltu.DM.01G048780.1	allene oxide synthase	12.87
Soltu.DM.04G028320.1	laccase	12.43
Soltu.DM.08G017780.2	Enoyl-CoA hydratase/isomerase family	12.38
Soltu.DM.09G024040.1	carboxyesterase	12.27
Soltu.DM.01G040940.1	terpene synthase	12.24
Soltu.DM.01G040950.1	terpene synthase	11.81
Soltu.DM.01G040930.1	terpene synthase	11.33
Soltu.DM.02G013170.1	FAD-binding Berberine family protein	11.26
Soltu.DM.03G018200.1	detoxifying efflux carrier	11.25
**18 hpi**	**Kuras**	**Down-regulated**
Soltu.DM.08G021790.1	lectin protein kinase family protein	-9.67
Soltu.DM.05G009830.1	cyclin-related	-9.10
Soltu.DM.03G016800.2	RNA-binding (RRM/RBD/RNP motifs) family protein	-8.68
Soltu.DM.11G026620.2	myb domain protein	-8.60
Soltu.DM.03G022230.7	thylakoid-associated phosphatase	-8.60
Soltu.DM.01G026120.2	chromatin remodeling	-8.59
Soltu.DM.03G030790.2	fatty acid desaturase	-8.18
Soltu.DM.05G007470.2	Sterile alpha motif (SAM) domain-containing protein	-7.94
Soltu.DM.04G027060.3	Inositol monophosphatase family protein	-7.73
Soltu.DM.12G023400.2	WLM domain containing protein	-7.70
**36 hpi**	**Kuras**	**Up-regulated**
Soltu.DM.06G033990.1	Transcription factor jumonji (jmj) family protein / zinc finger (C5HC2 type) family protein	11.92
Soltu.DM.06G023620.2	BURP domain-containing protein	10.19
Soltu.DM.09G014180.1	terpene synthase	10.13
Soltu.DM.08G005570.1	auxin response factor	10.03
Soltu.DM.03G034140.1	Pectinacetylesterase family protein	9.99
Soltu.DM.01G003520.1	serine-type endopeptidase inhibitors	9.93
Soltu.DM.08G028070.1	Lactoylglutathione lyase / glyoxalase I family protein	9.88
Soltu.DM.10G000900.1	copper ion binding;electron carriers	9.66
Soltu.DM.07G013680.2	Pyridoxal phosphate (PLP)-dependent transferases superfamily protein	9.49
Soltu.DM.05G002820.1	Glutathione S-transferase family protein	9.48
**36 hpi**	**Kuras**	**Down-regulated**
Soltu.DM.02G033100.4	shaggy-like kinase	-9.70
Soltu.DM.09G030690.1	Auxin-responsive family protein	-9.04
Soltu.DM.06G003240.4	thiaminC	-8.74
Soltu.DM.01G038650.1	SGNH hydrolase-type esterase superfamily protein	-8.59
Soltu.DM.02G031500.6	EXS (ERD1/XPR1/SYG1) family protein	-8.55
Soltu.DM.01G034500.2	conserved hypothetical protein	-8.20
Soltu.DM.02G013810.2	chlorophyll A/B binding protein	-8.14
Soltu.DM.08G009420.1	Tetratricopeptide repeat (TPR)-like superfamily protein	-8.11
Soltu.DM.07G014240.2	Cysteine proteinases superfamily protein	-7.94
Soltu.DM.08G029010.2	Leucine-rich repeat (LRR) family protein	-7.86

### Functional classification of DEGs

**Fig. 4:**
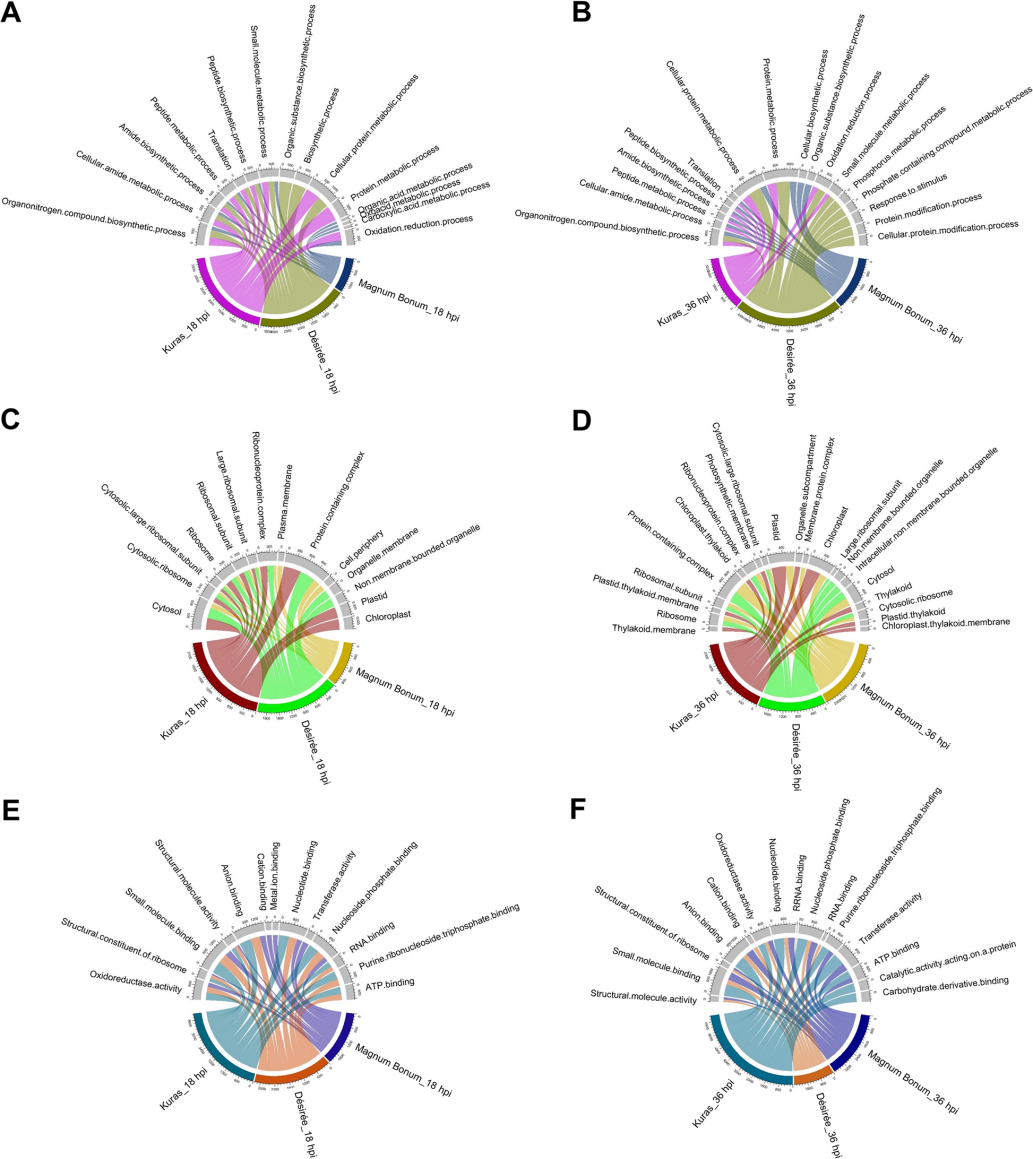
Gene Ontology (GO) terms enriched in DEGs. Top 10 GO terms enriched in **A**&**B** biological process (BP), **C**&**D** cellular components (CC), and **E**&**F** molecular function (MF) from different potato cultivars at 18 and 36 hpi, respectively

The Gene Ontology (GO) enrichment analysis was carried out with total as well as up- and down-regulated DEGs from different potato cultivars and time points. Analysis showed that many top functional categories of the biological process (BP), cellular components (CC), and molecular function (MF) were commonly over-represented across the different potato cultivars and time points (Fig. 4A-F). The commonly over-represented (FDR < 0.05) top 10 GO term for BP at 18 and 36 hpi was the Organonitrogen compound biosynthetic process (GO:1,901,566). Other GO terms such as Cellular amide metabolic process (GO:0,043,603), Peptide metabolic process (GO:0,006,518), Peptide biosynthetic process (GO:0,043,043), Small molecule metabolic process (GO:0,044,281), cellular protein metabolic process (GO:0,044,267) were enriched in either 18 or 36 hpi (Fig. 4A&B). For CC at 18 hpi, ribosome (GO:0,005,840) and cytosol (GO:0,005,829) were common between the three potato cultivars and there were no GO terms in common at 36 hpi (Fig. 4C&D). For MF, oxidoreductase activity (GO:0,016,491), small molecule binding (GO:0,036,094), anion binding (GO:0,043,168), and nucleotide binding (GO:0,000,166) were common GO terms present across the potato cultivar and time points (Fig. 4E&F). Apart from the common GO terms, there were unique functional categories specific for individual potato cultivars and time points. To gain further insight into the biological significance, up- and down-regulated DEGs from different potato cultivars and time points were analyzed which revealed that many of the top enriched GO terms from the total DEGs fall under the up-regulated DEGs category. One of the highly enriched BP GO terms in the down-regulated DEGs across the potato cultivar and time points were Photosynthesis (GO:0,015,979), except for Désirée 36 and Magnum Bonum 18 hpi (Additional file 5).

**Fig. 5:**
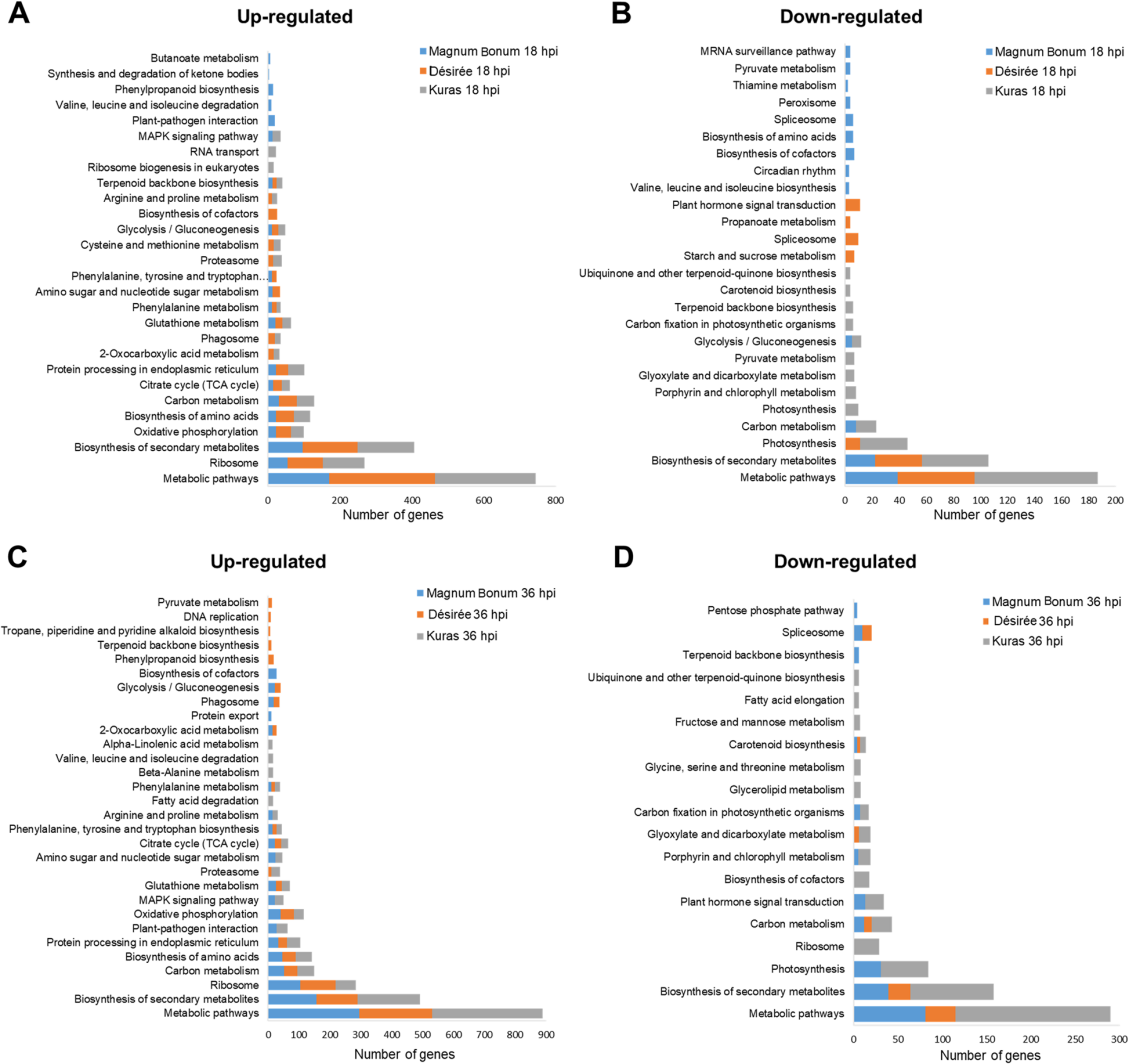
KEGG enrichment pathway analysis of DEGs. Top 20 enriched functional pathway categories of **A**&**B** up- and down-regulated DEGs at 18 hpi, respectively **C**&**D** up- and down-regulated DEGs at 36 hpi, respectively from Magnum Bonum, Désirée, and Kuras

The Kyoto Encyclopedia of Genes and Genomes (KEGG) pathway analysis was carried out to understand an overview of diverse pathways involved in the total as well as up- and down-regulated DEGs. The overall number of enriched pathways in different potato cultivars and time points are provided in Table 3. The metabolic pathways and biosynthesis of secondary metabolites were the commonly enriched pathways across the potato cultivars and time points. Pathways like the ribosome, carbon metabolism, protein processing in endoplasmic reticulum, biosynthesis of amino acids, citrate cycle (TCA cycle), and oxidative phosphorylation were significantly enriched at 18 hpi across the potato cultivars in the up-regulated DEGs. The number of transcripts in the enriched pathways was significantly higher in the up-regulated DEGs than in the down-regulated. The top 20 metabolic pathways enriched in each of the up- and down-regulated DEGs of different potato cultivars and at 18 and 36 hpi are shown in Fig. 5A&B and Fig. 5C&D, respectively. The complete list of GO and KEGG enrichment analyses of the DEGs is given in Additional file 5.

**Table 3:** The number of enriched pathways in KEGG analysis for the DEGs of different potato cultivars with *A. solani* infection. Different time points, Total DEGs, Up- and Down-regulated DEGs

**Potato cv**	**18 hpi**			**36 hpi**		
	**Total DEGs**	**Up-regulated**	**Down-regulated**	**Total DEGs**	**Up-regulated**	**Down-regulated**
Magnum Bonum	40	40	11	66	63	12
Désirée	64	64	8	57	52	8
Kuras	67	62	13	69	63	16

### Transcription factors expressed in response to *A. solani* infection

**Fig. 6:**
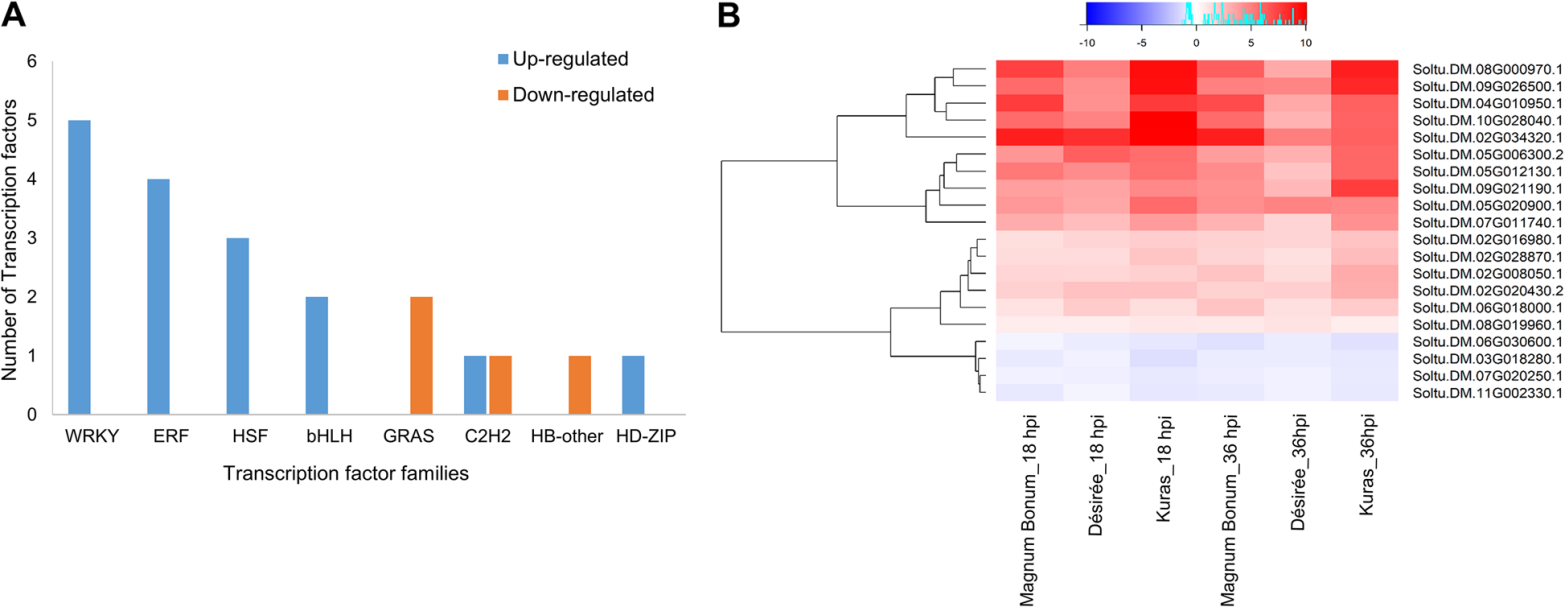
Commonly expressed TFs. **A** Up- and down-regulated TFs in different families, **B** Heat map showing the differential expression of TFs in different potato cultivars at 18 and 36 hpi

Several transcripts encoding transcription factors (TFs) were identified from the commonly expressed and unique DEGs. From the 649 commonly expressed DEGs, a total of 20 TFs encoding transcripts falling in eight families were identified, of which 16 were up-regulated (six families) and four were down-regulated (three families) (Fig. 6A). The WRKY (five) and ERF (four) TF families had the maximum number of transcripts that all were up-regulated. The expression patterns of the 20 identified commonly expressed TFs are present as a heat map in Fig. 6B, and the complete list is provided in Additional file 6. In the case of unique DEGs, an increasing trend in the TFs number was observed with increased susceptibility of potato cultivar to early blight and an increase in infection time, except for Désirée 36 hpi. At 18 hpi a total of 113, 145, and 436 TF transcripts were identified in Magnum Bonum, Désirée, and Kuras, respectively. Similarly, a high number of TFs was expressed in Kuras (160) followed by Magnum Bonum (76) at 36 hpi, while there was a decrease in TF number in Désirée (35) at 36 hpi. The identified TFs belong to approximately 42 families, and the dominant TF families identified were WRKY, ERF, bHLH, MYB, and C2H2 (Additional file 6).

**Fig. 7:**
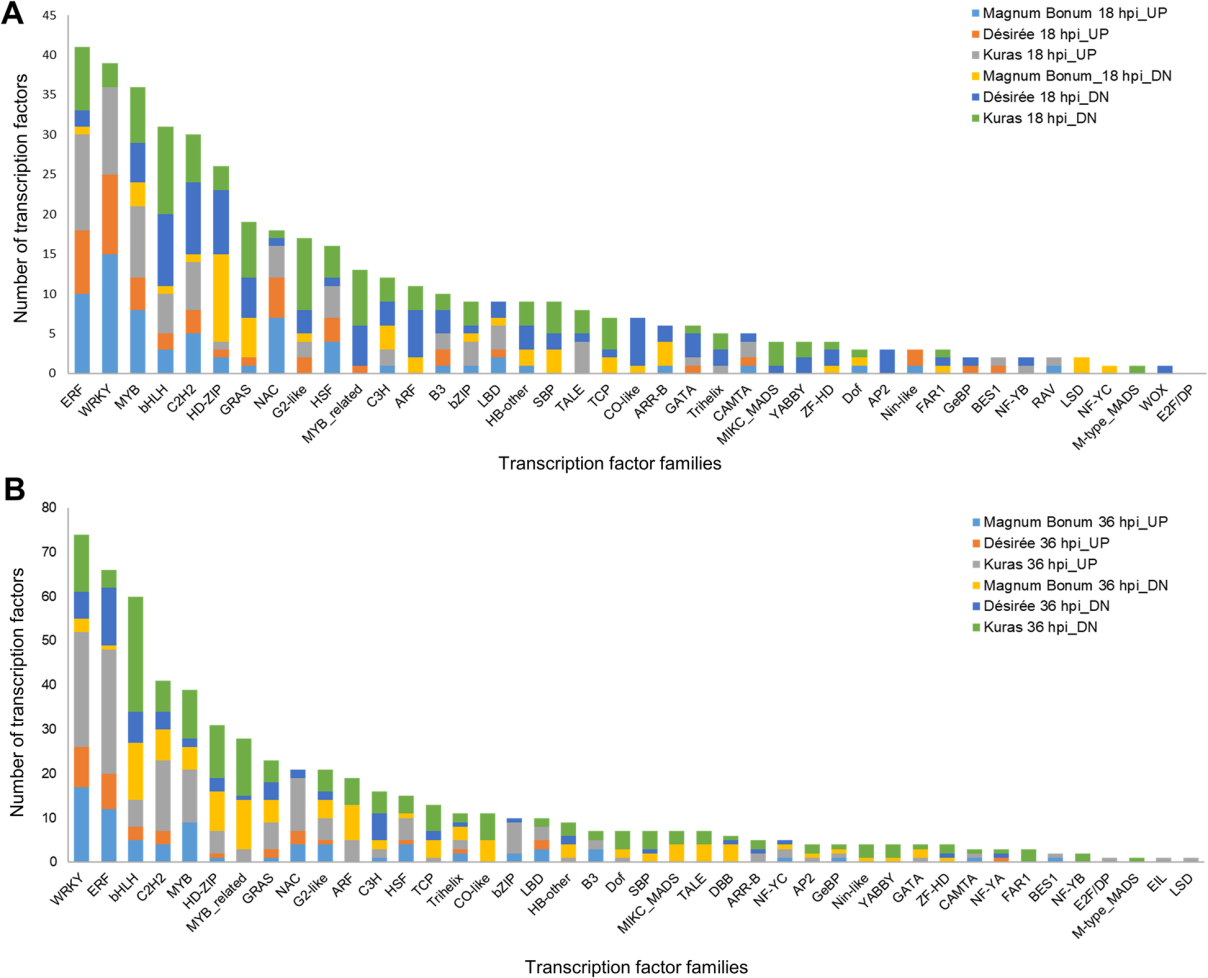
Number of TFs in different families identified from the up- and down-regulated DEGs of Magnum Bonum, Désirée, and Kuras at **A** 18 hpi and **B** 36 hpi

To further understand the expression pattern of these TFs, we analyzed the up- and down-regulated DEGs separately. Among all the up-regulated DEGs there was a significantly higher percentage (4.38%) of TFs in Magnum Bonum compared to Désirée (2.12%) and Kuras (2.73%) at the early time point (18 hpi) of infection, even though the later cultivars have a higher number of DEGs. There were a few differences in the TF percentage for the down-regulated DEGs between the 18 and 36 hpi in different potato cultivars (Additional file 6). We also observed significant differences in the number of TF transcripts expressed in each family between different potato cultivars, time points, and up- and down-regulated DEGs (Fig. 7A&B). In the up-regulated DEGs, the number of WRKY TFs identified in Magnum Bonum, Désirée, and Kuras at 18 hpi were 15, 10, and 11, respectively. The numbers were increased to 17, 9, and 26 for Magnum Bonum, Désirée, and Kuras, respectively, at 36 hpi. In the down-regulated DEGs, the WRKY transcripts were identified only in Kuras (three) at 18 hpi, and the numbers were three, six, and 13 for Magnum Bonum, Désirée, and Kuras, respectively, at 36 hpi. The other TF families with high numbers of genes at 18 and 36 hpi in the up-regulated DEGs were ERF, and NAC, which had fewer numbers in the down-regulated DEGs. Similarly, TF families like bHLH, HD-ZIP, GRAS, G2-like, MYB-related, and C3H were high in numbers for the down-regulated DEGs at 18 and 36 hpi. Also, a few TF families like SBP, ARF, TCP, Co-like, MIKC_MADS, TALE, and DBB were expressed only in the down-regulated DEGs at 18 and 36 hpi (Fig. 7A&B; Additional file 6).

### Metabolism pathway analysis of DEGs

We mapped DEGs to different functional categories by functional annotation based on Mercator4 and divisions in MapMan bins to understand the *A. solani* affected pathways in different potato cultivars with time points. In general, many genes coding for multiple components of the photosynthesis machinery, starch biosynthesis and degradation pathway were down-regulated in the susceptible cultivar Kuras compared to Désirée and Magnum Bonum. The down-regulation was higher at 36 hpi compared to 18 hpi. On the other hand, genes involved in the Jasmonic acid (JA) and Ethylene (ET) biosynthesis pathways, Mevalonate (MVA) pathway, Isoprenyll-PP, and Terpenes were up-regulated across the potato cultivars and time points with few exceptions. All the transcript ID and the fold change values are given in Additional files 7,8,9,10.

### Genes involved in photosynthesis

**Fig. 8:**
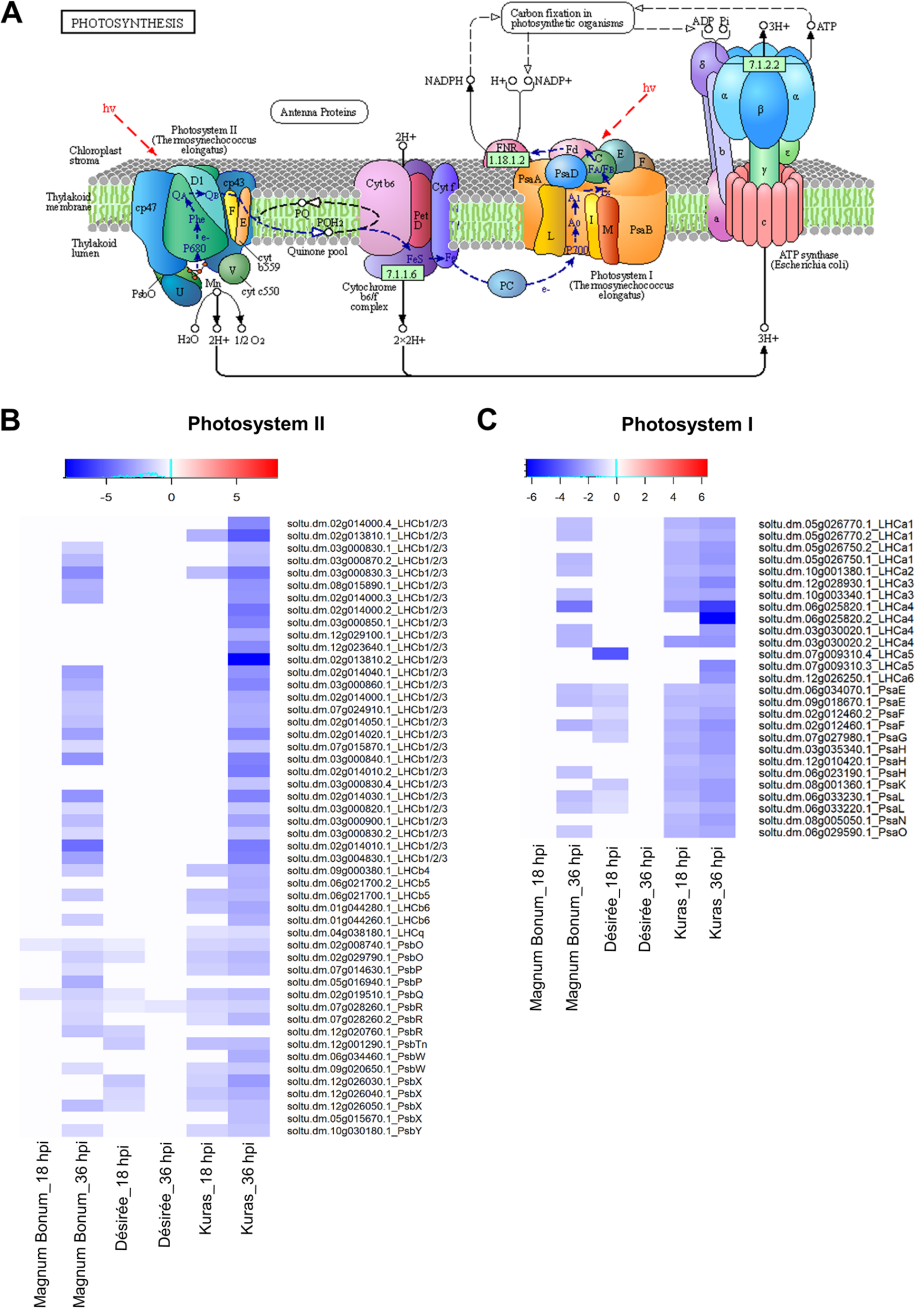
MapMan analysis of DEGs in photosynthesis. **A** KEGG pathway map for photosynthesis (1.2 energy metabolism—00,195, https://www.kegg.jp/kegg/pathway.html [89]), Heat map showing the expression pattern of different transcripts in **B** photosystem II and **C** photosystem I from Magnum Bonum, Désirée, and Kuras at 18 and 36 hpi

Photosynthesis is the key biochemical reaction occurring in all green plants. The genes involved in the light-harvesting complex (LHC), LHCa1, 2, 3, 4, 5, and 6 of the LHC1, PsaD, E, F, G, H, K, L, N, and O of the photosystem I (PS I) complex, and the high chlorophyll fluorescence (HCF) 101, PS I assembly 2 (PSA2), and the assembly factor PSA3 were highly down-regulated in Kuras at 18 and 36 hpi. Compared to Kuras, only a few of the above genes were down-regulated in Magnum Bonum 36 hpi and Désirée at 18 hpi; also, the extent of down-regulation was less (Fig. 8A&C; Additional file 7). Many genes involved in the LHCII, such as LHCb1/2/3, LHCb4, 5, 6, and LHCq were two-fold down-regulated in Kuras 36 hpi compared to Magnum Bonum 36 hpi (Fig. 8A&B; Additional file 7). Similarly, genes of the PS II assembly (LPA2, LPA3, HCF 136, 173, 243, Psb27, 28, 32, 33, PAM68, LHC related protein (OHP1), psbJ/psbN-translation activator (LPE1), Thioredoxin (TRX-M), LHCII-stabilizing factor (SEP3) were down-regulated in Kuras at 18 and 36 hpi (Additional file 7). The components of the PS II oxygen-evolving center (PsbO/OEC33, PsbP, PsbQ, PsbR, PsbTn, PsbW, PsbX, PsbY), subunits and components of chlororespiration, components of cytochrome b6/f complex, plastocyanin electron carrier, PGRL1 of electron flow PGR5-PGR1 complex, Ferredoxin (Fd) targeted to NADP reduction, ATP synthase, and subunits of rubisco were down-regulated in Kuras at 18 and 36 hpi, as compared to Désirée and Magnum Bonum (Additional file 7).

### Genes involved in starch biosynthesis and degradation

The genes involved in starch biosynthesis were down-regulated in Kuras at 18 and 36 hpi. The maximum of 1.5 and 2.2 fold down-regulation was observed with a large subunit of ADP-glucose pyrophosphorylase gene (soltu.dm.01g024440.1) in Kuras at 18 and 36 hpi, respectively. On the contrary, no starch biosynthesis genes were down-regulated at 18 hpi in Magnum Bonum, and starch synthase (SS) III (soltu.dm.02g020170.2) was the only gene down-regulated at 36 hpi in Magnum Bonum. At 18 hpi in Désirée, large subunit APL of ADP-glucose pyrophosphorylase (soltu.dm.01g024440.1), starch branching enzyme (soltu.dm.09g004100.1), and component ISA1 of ISA1-ISA2 isoamylase heterodimer (soltu.dm.07g005540.1) were down-regulated and starch synthase (SS) III (soltu.dm.02g020170.2) and scaffold protein of amylose biosynthesis (soltu.dm.02g026830.2) were down-regulated at 36 hpi. Similarly, genes such as alpha-amylase-binding scaffold protein (LSF1) (soltu.dm.12g016610.2) and beta-amylase (soltu.dm.07g018100.1) were down-regulated (1.6 fold) in Kuras at 18 hpi and a 1.85 fold down-regulation was observed for the plastidial alpha-glucan phosphorylase (PHS1) (soltu.dm.05g000570.1) gene at 36 hpi. Only a few genes were down-regulated in Magnum Bonum and Désirée at 18 and 36 hpi (Additional file 8).

### Genes involved in the mevalonate pathway, isoprenyl-PP, and terpenes

The genes involved in the mevalonate (MVA) pathway, Isoprenyl-PP, and Terpenes were up-regulated in all the potato cultivars and time points with a few exceptions (Additional file 9). All the seven enzymes (acetyl-CoA C-acyltransferase, 3-hydroxy-3-methylglutaryl-CoA synthase, 3-hydroxy-3-methylglutaryl-CoA reductase, mevalonate kinase, phosphomevalonate kinase, mevalonate diphosphate decarboxylase, and isopentenyl diphosphate isomerase) involved in the MVA pathway were up-regulated in different potato cultivars and time points. The maximum up-regulation of these transcripts was observed in Kuras at 36 and 18 hpi followed by Désirée and Magnum Bonum. One of the transcripts for the enzyme acetyl-CoA C-acyltransferase (soltu.dm.07g015120.1) was up-regulated in all the potato cultivars and another transcript soltu.dm.04g010070.3 for the same enzyme was expressed only in Désirée 36 hpi with a fold increase of 8.36. The 3-hydroxy-3-methylglutaryl-CoA synthase (soltu.dm.08g026810.1) was up-regulated 4.86 and 7.84 fold in Kuras at 18 and 36 hpi and 3.69 fold in Magnum Bonum 36 hpi (Additional file 9). The enzymes farnesyl diphosphate (FDP) synthase and isoprenyl diphosphate synthase (IDS) were up-regulated, and FDP synthase maximum fold-change (7.07) was observed at 36 hpi in Kuras, followed by Désirée at 18 hpi (4.07 fold). The IDS was up-regulated in all potato cultivars and time points, except Magnum Bonum at 36 hpi (Additional file 9). Many of the transcripts code for mono-/sesquiterpene-/diterpene synthases were up-regulated in multiple potato cultivars. The mono-/sesquiterpene-/diterpene synthases transcripts (soltu.dm.01g040930.1, soltu.dm.01g040950.1, soltu.dm.07g017540.1, soltu.dm.01g040960.1 and soltu.dm.07g017580.1) were expressed in all potato cultivars at 18 and 36 hpi. The highest fold-change expression of 11.81 and 11.33 was observed for soltu.dm.01g040950.1 and soltu.dm.01g040930.1 in Kuras 18 hpi. A down-regulation (7.90 fold) was noticed for the transcript soltu.dm.07g017230.1 in Désirée 36 hpi (Additional file 9).

### Genes involved in JA and ET biosynthesis pathways

**Fig. 9:**
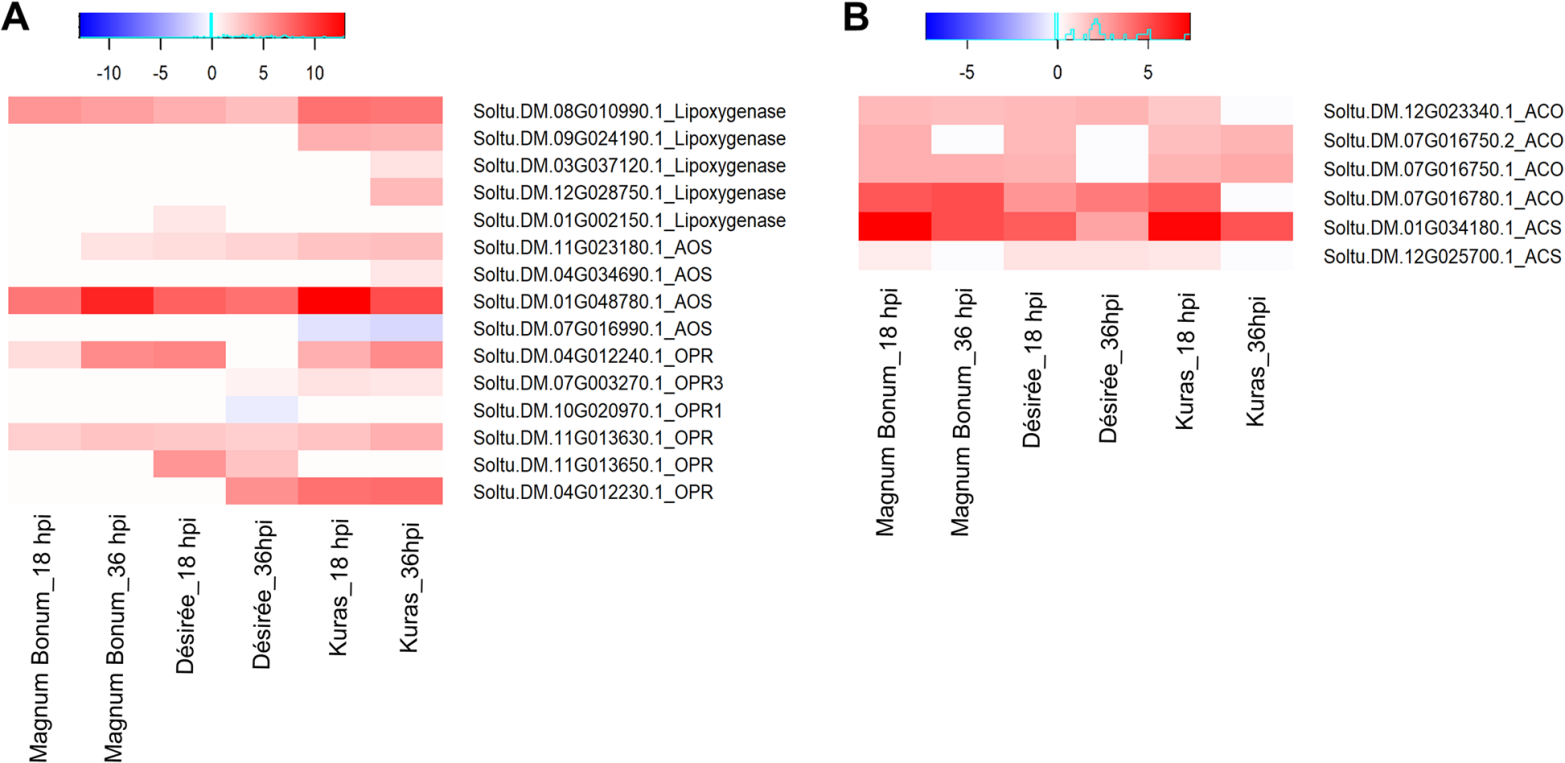
Heat map showing the expression pattern of different transcripts identified from Magnum Bonum, Désirée, and Kuras at 18 and 36 hpi. **A** JA biosynthetic pathway, **B** ET biosynthetic pathway

The JA and ET biosynthesis and signaling are shown to be very critical for imparting plant resistance against necrotrophic pathogen attacks [23, 24]. We identified multiple transcripts encoding the enzymes involved in the JA and ET biosynthesis pathways; as expected, all were up-regulated, except for two (Fig. 9; Additional file 10). The JA biosynthesis enzyme, Lipoxygenase (LOX; Soltu.DM.08G010990.1) was most up-regulated in Kuras 18 and 36 hpi (7.12 and 6.73 fold, respectively) followed by Magnum Bonum and Désirée. The Allene oxide synthase (AOS; Soltu.DM.01G048780.1) had the highest expression in Kuras 18 hpi (12.87 fold) and reduced to 8.92 fold at 36 hpi and for Magnum Bonum, the AOS levels were increased at 36 hpi (10.95 fold) compared to 18 hpi (6.93 fold). The 12-oxophytodienoate reductase (Soltu.DM.04G012240.1) levels increased at 36 hpi for Magnum Bonum and Kuras, but the maximum fold-change was seen in Désirée at 18 hpi (6.09 fold) (Additional file 10). Another transcript encodig a 12-oxophytodienoate reductase (Soltu.DM.04G012230.1) was 7.01, 7.29, and 5.56 times up-regulated in Kuras at 18 and 36 hpi and Désirée 36 hpi, respectively, and was not detected in Magnum Bonum at 18 and 36 hpi and Désirée at 18 hpi. Similarly, multiple transcripts were identified for 1-aminocyclopropane-1-carboxylate synthase (ACS) and 1-aminocyclopropane-1-carboxylate oxidase (ACO) part of the ET biosynthesis. Both ACS and ACO were significantly up-regulated in all potato cultivars and time points. One of the transcripts for ACS (Soltu.DM.01G034180.1) was highly up-regulated at 18 hpi in all potato cultivars and reduced at 36 hpi. The ACO transcript (Soltu.DM.07G016780.1) fold-change was maximum (4.98) in Magnum Bonum at 36 hpi followed by 18 hpi (4.79), and Kuras showed a 4.45 fold-increase at 18 hpi (Additional file 7).

## Discussion

There is a limited understanding of the molecular mechanism and the factors involved in potatoes early blight disease development due to a limited number of studies. Since no complete genetically resistant potato sources have been identified against early blight, more studies are required with partially resistant cultivars to understand the disease resistance mechanisms. In a recent study, transcriptome analysis was carried out in leaves of potato cultivar Désirée inoculated with *A. solani* (sequenced strain NL03003; CBS 143,772) [18, 25]. The study focused on capturing the early molecular changes occurring at the transcriptome level in potato leaves at the time of *A. solani* appressorium formation and penetration and later responses to necrosis. In the current study, we generated transcriptomes at two time points, 18 and 36 hpi, from three potato cultivars with significant differences in the necrotic lesions sizes (Fig. 1). Magnum Bonum was found to be the most resistant potato cultivar, followed by Désirée and Kuras consistent with Odilbekov et al. [11, 17].

The transcriptome profiling revealed significant differences in the number of DEGs in the different potato cultivars and time points. The early blight-susceptible potato cultivar Kuras had 4046 DEGs at 18 hpi followed by Désirée (3398) and Magnum Bonum (2052). At 36 hpi DEGs increased to 6261 for Kuras and 3753 for Magnum Bonum but decreased to 2440 in Désirée (Fig. 2; Additional file 1). A reduction in the number of DEGs in the Désirée cultivar at later time points has also previously been reported by Brouwer et al. [18], who identified 1859 DEGs at 24 hpi followed by a decrease to 572 DEGs at 48 hpi in Desiree. Also, there was a significant difference in the total number of DEGs identified with the Désirée cultivar at 24 hpi in the Brouwer et al. [18] study as compared to 18 hpi in the current study. Apart from the difference in time point, we speculate that the difference in the number of DEGs may be due to the different versions of the reference genome (PGSC_v4.03 and DM 1–3 516 R44 v6.1) used for mapping the reads or the difference in the virulence of the *A. solani* strain used for inoculations. In a comparative study between NL03003 and As112 *A. solani* strains, we could identify that the latter had more conidia formation and higher virulence capacity (data not shown). Further studies are required to find whether this is a reason.

TFs are central regulators of gene expression and plant defense signaling in response to various biotic stresses [26–28]. TFs interact with multiple downstream targets through sequence-specific binding with the cis-elements of gene promoters [29, 30]. Since resistance and susceptibility of the host plants depend on the speed and level of expression of immune response pathway genes, TFs significantly influence plant defense. WRKY, bHLH, AP2/ERF, C2H2, bZIP, NAC, MYB, HD-ZIP, G2-like, HSF, ARF, and GRAS are well-known TF families involved in defense-related gene expression against various pathogens [31–33]. In the present study, many transcripts were identified from most of these TF families. WRKY and ERF are the two top TF families expressed, and most of these transcripts were up-regulated in all potato cultivars at 18 and 36 hpi (Fig. 7A&B; Additional file 6). WRKY and ERF TFs are reported to be very important in plant-pathogen interactions and impart resistance to fungal pathogens [34, 35]. WRKY TFs contain a ‘WRKYQK’ domain that can regulate several signaling pathways, including histone deacetylases, MAP kinases, and phytohormones [36], and are also involved in the secondary metabolite biosynthesis [37]. Similarly, ERFs were reported to be induced by pathogens, ethylene, JA, and regulate the expression of downstream pathogenesis-related genes [38, 39]. It has been shown that overexpression of WRKY and ERF TFs resulted in increased resistance against fungal pathogens [40, 41]. Similarly, it is well known that the expression of a few TF families can increase susceptibility to fungal pathogens, and one among them is the homeodomain-leucine zipper (HD-ZIP). The overexpression of GhHB12, an HD-ZIP TF in cotton, increased the susceptibility to fungal pathogens *Botrytis cinerea* and *V. dahlia* [42]. We also noticed that the majority of the HD-ZIP class TFs expression were down-regulated in the resistant cultivar Magnum Bonum compared to the susceptible cultivar Kuras at 18 hpi. A few more groups of TF families with differential expression in partially resistant and susceptible potato cultivars were identified (Additional file 6). These may lead to the improved resistance exhibited by the Magnum Bonum cultivar, and to confirm this, additional overexpression and silencing studies are required.

It is well documented that the pathogen attack can decline the net photosynthetic rate and induce carbon starvation in sink tissues [43–45]. In the current study, KEGG enrichment analysis with the down-regulated DEGs showed a significant enrichment with photosynthesis functional category in the susceptible cultivar Kuras at 18 and 36 hpi, which was not the case for Magnum Bonum and Désirée. Out of the total 75 photosynthesis genes, 34 and 53 were down-regulated in Kuras at 18 and 36 hpi, respectively, and in Magnum Bonum 31 genes were down-regulated at 36 hpi **(**Additional file 5**)**. Most genes coding for proteins in PSI and PSII reaction centers, several elements of the LHC associated with PSI and PSII, components of cytochrome b6/f complex, ATP synthase, and subunit of rubisco were significantly down-regulated (Fig. 8A-C; Additional file 7**)**. The down-regulation of photosynthesis helps to reallocate the resources toward plant defense mechanisms against pathogen attack [46]. This is supported by the current study’s up-regulation of many defense signaling pathways and genes involved in the plant defense responses.

The majority of the top differentially expressed transcripts at 18 and 36 hpi in Magnum Bonum and Kuras were involved in plant defense responses (Table 2). The peroxidase superfamily proteins are involved in various physiological processes, including active host plant defense responses against pathogens, cell wall lignification, and oxidative stress [47–51]. We noticed multiple peroxidase superfamily proteins (Soltu.DM.10G018980.1; Soltu.DM.10G019020.1) were in the top 10 up-regulated transcripts at 18 hpi in Magnum Bonum and Kuras. The up-regulation was higher for the susceptible potato cultivar Kuras compared to Magnum Bonum (Table 2). Similarly, plants produce several secondary metabolites in response to various environmental cues, including biotic and abiotic stresses [52]. These include simple hydrocarbon terpenes and terpenoids or isoprenoids that are produced via the activation of the cytosolic MVA pathway. Naets et al. [53] showed a strong negative correlation between the up-regulation of MVA pathway at early time points with the success of *B. cinerea* infection. From the current study, we identify that all the transcripts involved in the MVA pathway were up-regulated and support the fact that terpenoids are key for plant defense. The terpenoids are a large and structurally diverse class of terpenes synthesized from the precursor’s geranyl pyrophosphate (GPP), farnesyl pyrophosphate (FPP), and geranylgeranyl pyrophosphate (GGPP) by terpene synthases [54–56]. The involvement of terpene synthase is indispensable for the synthesis of diverse volatile or semi-volatile and non-volatile terpenoids that are emitted in response to pathogen attacks and function directly as defensive phytoalexins [57–59]. This was supported by the up-regulation of multiple transcripts of terpene synthase in Magnum Bonum and Kuras at 18 and 36 hpi (Table 2; Additional files 1,2,3, and 9).

The carboxylesterases (CXEs; EC 3.1.1.1) are hydrolases and members of the α/β-hydrolase fold superfamily, which comprises a large group of enzymes, such as proteases, lipases, esterases, and transferases [60, 61]. They are reported to be associated with plant-pathogen interaction and hypersensitive responses [62, 63]. The transgenic *Arabidopsis* plants overexpressing *AtCXE8* showed better resistance to *Botrytis cinerea* [64]. Some of the CXEs in *Vitis flexuosa* were up-regulated in response to *Botrytis cinerea* and *Rhizobium vitis* infection, suggesting a putative role in the defense mechanism during pathogen infection [63]. A carboxylesterase (Soltu.DM.09G024040.1) was the top gene expressed at 36 hpi in Magnum Bonum and 18 hpi in Kuras in the current study. Interestingly, carboxylesterase and AOS transcripts expressed in Magnum Bonum at 36 hpi were identified at 18 hpi in Kuras with higher Log2 fold-change. Also, the detoxifying enzyme family proteins like detoxifying efflux carrier (DTX; Soltu.DM.03G018200.1) and Glutathione S-transferase (Soltu.DM.05G002820.1) were identified in Kuras top 10 up-regulated transcripts at 18 and 36 hpi, respectively. Even though there was a higher fold change expression of genes in Kuras than other cultivars studied, the former was susceptible to early blight and the exact reason for susceptibility needs to be further investigated.

The signaling and cross-talk between JA, ET, and Salicylic acid (SA) are well documented for imparting resistance against various biotrophic and necrotrophic pathogens [65–68]. The activation of a specific hormonal pathway depends on the nature of the pathogen and the host plant. In general, it is believed that JA and ET signaling is important for resistance against necrotrophic pathogens and SA signaling for biotrophs [69, 70]. Sivasankar et al. [71] showed that ET could induce AOS expression, a rate-limiting enzyme in JA biosynthesis. Similarly, MeJA can enhance the expression of ACO, resulting in increased ET synthesis [72]. Both JA and ET work synergistically, and their signaling pathways are interlinked [72, 73]. In this study, transcripts for most of the enzymes involved in both JA and ET biosynthesis were highly up-regulated. The maximum expression levels were observed for AOS (Soltu.DM.01G048780.1) and ACO (Soltu.DM.07G016780.1) (Fig. 9; Additional file 10), pointing toward the fact that JA and ET biosynthesis pathways are interlinked and can act synergistically.

It has been reported that intact SA signaling is required for early blight disease resistance in potatoes [22]. However, we could not find a significant change of expression of genes related to the SA biosynthesis pathway after *A. solani* infection in the present study. The SA biosynthesis and signaling-associated transcripts were not detected in Magnum Bonum and Désirée. The only SA biosynthesis related transcript identified in Kuras was isochorismate synthase (ICS) (Soltu.DM.06G026140.1) which was down-regulated at 18 and 36 hpi. More recently, Brouwer et al. [18] showed that ET biosynthesis-related transcript 1-aminocyclopropane carboxylic acid oxidase 2 and transcripts encoding for JA biosynthesis, LOX and AOS were down-regulated in Désirée at 12 and 24 hpi. Contradictory results were observed in the current study with most of the transcripts coding for different ET and JA biosynthesis significantly up-regulated in Désirée and the other potato cultivars at 18 and 36 hpi (Fig. 9; Additional file 10). Our data suggest that JA and ET signaling pathways are also critical for resistance against the necrotrophic pathogen *A. solani*. In *Arabidopsis thaliana* the activation of the JA/ET signalling pathway leads to the upregulation of defense-related genes in response to necrotrophic pathogens [70, 74, 75], although, there are a few exceptions [73, 76].

## Conclusions

In this study, we analyzed the transcriptome changes in three potato cultivars with differences in the early blight resistance at two time points post *A. solani* infection. The DEGs identified from each potato cultivar and time point shed light on the molecular mechanism and factors operating towards partial resistance or susceptibility against early blight. Interestingly, a few of the top expressed transcripts in the partially resistant and susceptible cultivar, Magnum Bonum and Kuras, respectively, were similar; the latter had a higher Log2 fold expression but was more susceptible to *A. solani*. We were able to identify unique TFs expressed in specific potato cultivars at different time points. These TFs are potential new molecular targets and can influence the difference in global and specific gene expression observed. Many key up- and down-regulated transcripts identified from this study might be attractive targets for improved early blight resistance in potato. That different *A. solani* isolates trigger different responses in gene expression of the cultivar Désirée as well as the difference in response between the potato cultivars in this study raise the question of both strain- and cultivar-specific defense responses. This emphasises the importance of screening several cultivar-strain combinations to get a more comprehensive picture of host plant resistance/susceptibility.

## Methods

### Plant establishment and growth conditions

In vitro plants of three potato cultivars with different levels of early blight resistance previously determined in an in-house study Magnum Bonum (partially resistant), Désirée (moderately susceptible), and Kuras (susceptible compared to Désirée), were grown in 0.5 L pots (9 × 9x9.5 cm) filled with the potting mix (Exclusiv Blom and Plantjord, Emmaljunga Torvmull AB, Sweden) for 2 weeks and subsequently transferred to 2 L pots with the same potting mix and allow to grow for four more weeks. The plants were grown in a controlled environment in an artificial light chamber (160 µmol/s/m^2^, 16 h light and 8 h dark regime at 25 and 22 °C day and night temperatures, RH = 55–60%; Biotron, Alnarp, Sweden). The plants were watered twice a week.

### Fungal pathogen maintenance and culture preparation

*Alternaria solani* (strain As112), isolated from a naturally infected potato field in Sweden [11], was maintained in a 20% potato dextrose medium (PDA). For fresh culture plates, actively growing mycelial agar discs (5*5 mm) were placed on a PDA plate and incubated at room temperature in the dark for three days. Subsequently, plates were transferred to a UV-C light incubator (model OSRAM HNS15G13) programmed for an 8 h illumination with a dominant wavelength of 254 nm and a temperature of 18 °C to improve sporulation. The plates were incubated for 8 to 10 days, and conidia were harvested by flooding the plates with 10 mL of autoclaved tap water containing 0.01% (v/v) Tween 20 (Sigma Aldrich). The concentration of conidia was adjusted to 25,000 conidia/mL using a Fuchs Rosenthal hemocytometer counting chamber and immediately utilized for inoculation.

### Artificial fungal inoculation and disease assessment

After six weeks of growth, the three cultivars were randomized with four potato plants per incubation trolley. For inoculation, three individual potato plants were used from each cultivar with two leaves each from the center, around 22 to 25 days old. A maximum of three 15 µl droplets of inoculum carrying 25,000 conidia/mL of As112 was placed on the adaxial side on either side of the midrib of each leaflet, leaving the first two and the center leaflets. For control plants (hereafter mentioned as mock inoculation), 15 µl each of sterile water containing 0.01% (v/v) Tween 20 was used. Inoculations were adjusted so that the lights were turned off immediately after the inoculation. All trolleys were covered with plastic foils to maintain high humidity (> 95%) for the first 24 h to have an efficient infection. The trolleys were kept in the artificial light chamber under the conditions mentioned earlier. Three separate sets of plants were maintained for disease assessment and collecting the samples at 18 and 36 h time points for RNA sequencing following similar growth and inoculation conditions. Leaf disc samples for RNA sequencing were collected using an 8 mm diameter cork borer, including the inoculation spot in a 15 mL centrifuge tube flash-frozen in liquid nitrogen and stored at -80 °C until further use. Samples were collected at light hours from the inoculated and mock-inoculated leaflets at 18 and 36 hpi. Each plant was seen as a biological replication in the experiment. Plants were kept for an additional four days to carry out a disease assessment when evident lesions had appeared by measuring the diameter of the necrotic spot with a ruler.

### Sample processing, RNA extraction, and quality assessment

Leaf disc samples were homogenized using (FastPrep®-24, Classic (MP Biomedicals, USA) high-speed benchtop tissue homogenizer at 5.0 m/s for 60 s (repeated three times), and 100 mg tissue per sample was transferred to a 1.5 mL microcentrifuge tube. Total RNA was extracted using the RNeasy Plant Mini kit (Qiagen, Hilden, Germany) according to the manufacturer’s protocol. An added DNase treatment step was performed on the column using the PureLink™ DNase set (ThermoFisher Scientific, Massachusetts, USA) according to the manufacturer’s protocol. The RNA concentration and purity were estimated by spectrophotometer using a NanoDrop ND-1000 (Waltham, MA, USA), and the RNA integrity number (RIN) was assessed with the Agilent 2100 Bioanalyzer (Agilent Technologies, CA, USA). Samples with RIN values 8.0 or above were used for library preparation.

### Library preparation and transcriptome sequencing

TruSeq Stranded mRNA Sample Prep Kit (Illumina) was used for the library preparation. Briefly, polyadenylated messenger RNA (mRNA) was captured from total RNA per sample using poly-T oligo beads and fragmented. Using the random primers and reverse transcriptase, RNA fragments are copied into the first strand and subsequently to second-strand cDNA. The cDNA samples were end-repaired, phosphorylated, and polyadenylated before the ligation of TruSeq adaptors with sample-specific barcode sequences for multiplexing. Fragments containing TruSeq adapters at both ends were selectively enriched with PCR, and the quality and quantity of the enriched libraries were assessed. Paired-end (150 bp) mRNA reads were generated from three biological replicates samples (18 and 36 hpi) using the Illumina NovaSeq6000 S4 sequencing platform (SciLifeLab, Stockholm, Sweden). All raw sequencing data generated in this study have been deposited in National Center for Biotechnology Information (NCBI) under the BioProject accession number PRJNA867676.

### Read processing, Mapping, and DEG analysis

Raw read quality control (QC) check was performed with FastQC v0.11.7 [77], and multiple sample visualization MultiQC v1.6 [78] tool was used. Initial filtering steps were performed to remove ribosomal RNAs (rRNAs) by aligning reads with SILVA and rfam databases using Sortmerna-v2.1b [79] tool, and all TruSeq3 adapters were trimmed with the Trimmomatic-v0.36 [80] setting MINLEN:20 in bases and SLIDINGWINDOW:5:20 with other default parameters. The second round of QC checks was performed on independent samples using the same tools mentioned above. The whole genome of DM 1–3 516 R44 v6.1 assembly (http://spuddb.uga.edu/dm_v6_1_download.shtml) was used for reference alignment. The mRNA reads were aligned to the genome using the splice aligner STAR-v2.5.4a [81] tool with, –twopassMode Basic, –sjdbGTFfeatureExon CDS, –outReadsUnmapped Fastx, keeping other parameters as default. Transcript abundance was estimated with Salmon v1.3.0 [82]. Raw read counts were used for Differential Expression (DE) analysis with DESeq2 [83, 84], and in-built “Relative Log Expression” (RLE) [85] normalization was performed. The BLAST search was performed to get the gene coordinates from the alternative potato reference genome (PGSC_DM_v4.03) [86]. PCA analysis was carried out to visualize the variation of the samples used for expression analysis using the R package DESeq2 v1.16.1. The PCA plot was developed by the plotPCA function with the rlogTransformation (regularized-logarithm transformation) for clustering. The differential expression analysis was performed between the *A. solani* inoculated samples with the mock-inoculated samples at a specific time point. We used a false discovery rate (FDR) < 0.05 set as the threshold for significant differential expression without considering the absolute log2 (fold change) cut-off value. For visualization, Venn diagrams were created using an online tool (http://bioinformatics.psb.ugent.be/webtools/Venn/) and an R package version 1.6.20 venn.

### Gene Ontology, Metabolic pathway enrichment, and Transcription factor analysis of DEGs

To obtain further insight into different metabolic processes and functional enrichment analysis, including GO and KEGG pathways, we used ShinyGO v0.741 [87] with default parameters, and an FDR < 0.05 cut-off was used to identify significantly enriched GO terms. The top-10 enriched GO terms from the BP, CC, and MF were visualized in a chord diagram created using the R package circlize [88]. Similarly, the top-20 enriched KEGG pathways for up- and down-regulated DEGs at 18 and 36 hpi between the three potato cultivars were also visualized in a bar plot. KEGG pathway database was used to represent the photosynthesis pathway map (https://www.kegg.jp/kegg/pathway.html [89]). Furthermore, to explore the expression of different families of TFs during the *A. solani* infection, the protein sequences of the DEGs were extracted from DM_1-3_516_R44_potato.v6.1.working_models.pep.fa. and searched against the Plant Transcription Factor Database v5.0 (PlantTFDB v5.0; http://planttfdb.cbi.pku.edu.cn) [90] across all the potato cultivars and time points.

### MapMan metabolic analysis

The DEGs were mapped to the metabolic pathway using MapMan (version 3.6.0R1 https://mapman.gabipd.org/home) [89, 91]. Since MapMan software lacks the mapping file for the DM 1–3 516 R44 v6.1 potato genome, we generated the corresponding mapping file using Mercator v4.0 (http://www.plabipd.de/portal/mercator-sequence-annotation) by uploading all the predicted protein sequences of the DM 1–3 516 R44 v6.1 potato genome (DM_1-3_516_R44_potato.v6.1.working_models.pep.fa.). The mapping file was downloaded and imported into MapMan, and the latest pathway files starting with X4.2 were downloaded from the MapMan store to analyze the metabolic regulation of DEGs.

### Statistical analysis

Data were analyzed using SPSS 20.0 Software (SPSS Inc., Chicago, IL, USA). We used One-way ANOVA followed by post-hoc Tukey HSD analysis to test the significance of disease severity between the potato cultivars (**p value < 0.01 and *p value < 0.05).

## Supplementary Information

**Additional file 1.** 

**Additional file 2.** 

**Additional file 3.** 

**Additional file 4.** 

**Additional file 5.** 

**Additional file 6.** 

**Additional file 7.** 

**Additional file 8.** 

**Additional file 9.** 

**Additional file 10.** 

**Additional file 11.** 

## Data Availability

The data generated for this study can be found in NCBI (https://www.ncbi.nlm.nih.gov/nuccore/) BioProject database under accession number PRJNA867676.

## Abbreviations

RNA-Seq: RNA Sequencing
RIN: RNA integrity number
QC: Quality control
rRNAs: Ribosomal RNAs
hpi: Hours post-infection
PDA: Potato dextrose medium
DE: Differential Expression
RLE: Relative Log Expression
DEGs: Differentially expressed genes
FDR: False discovery rate
GO: Gene Ontology
KEGG: Kyoto Encyclopedia of Genes and Genomes
PCA: Principal component analysis
TFs: Transcription factors
MVA: Mevalonate
QTL: Quantitative trait loci
SA: Salicylic acid
JA: JAsmonic acid
ET: Ethylene
LHC: Light harvesting complex
HCF: High chlorophyll fluorescence
PS: Photosystem
PSA: Photosystem assembly
FDP: Farnesyl diphosphate
IDS: Isoprenyl diphosphate synthase
LOX: Lipoxygenase
AOS: Allene oxide synthase
ACS: 1-Aminocyclopropane-1-carboxylate synthase
ACO: 1-Aminocyclopropane-1-carboxylate oxidase
CXEs: Carboxylesterases
GPP: Geranyl pyrophosphate
FPP: Farnesyl pyrophosphate
GGPP: Geranylgeranyl pyrophosphate
