# Supplementary material for: Comprehensive transcriptome analysis of different potato cultivars provides insight into early blight disease caused by Alternaria solani
Source: BMC Plant Biol. 2023 Mar 8;23:130. doi: 10.1186/s12870-023-04135-9 (PMC9993742; doi:10.1186/s12870-023-04135-9)
Supplement: Supplementary file 11 — Additional file 11. [file 12870_2023_4135_MOESM11_ESM.pdf]

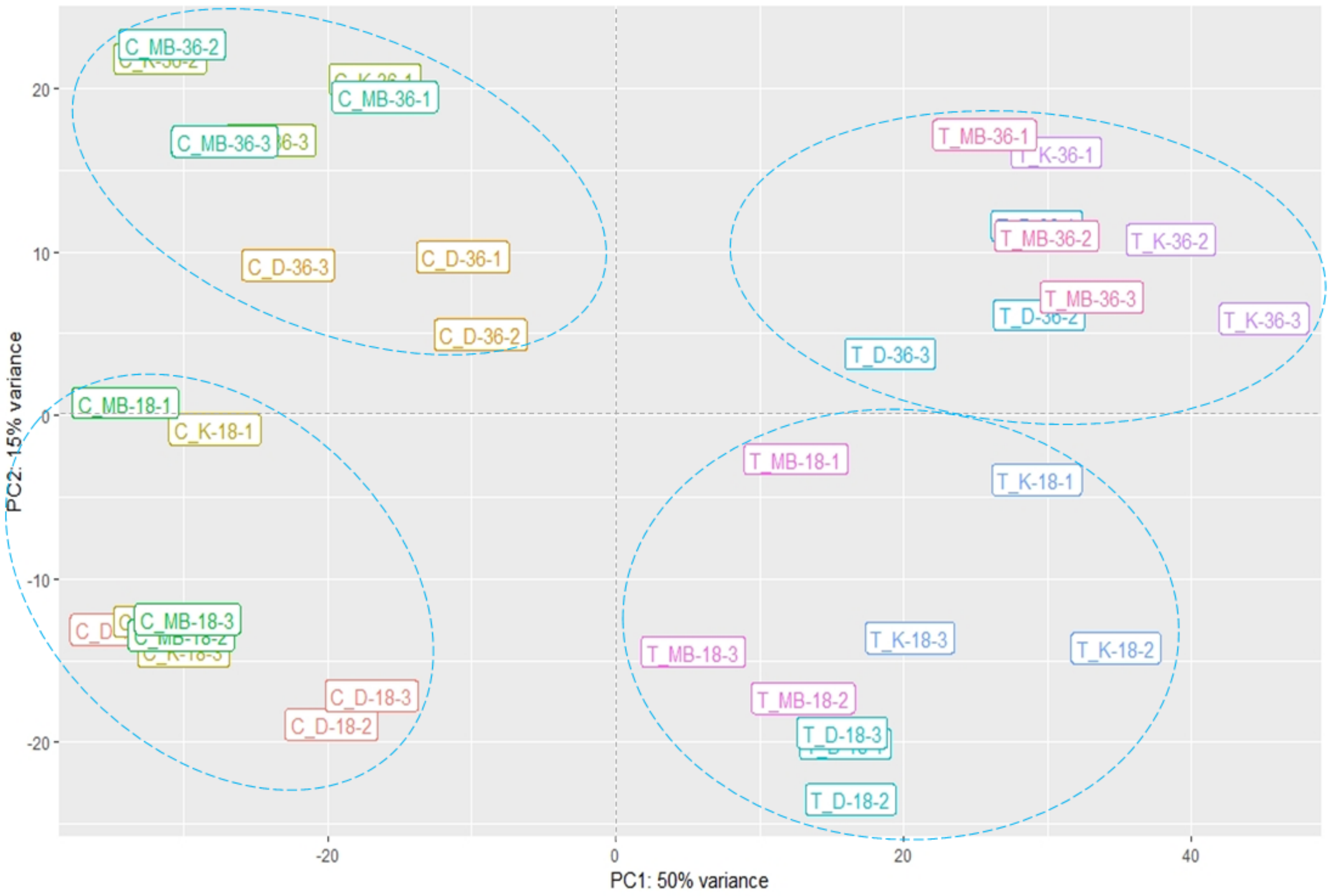

Figure S1: PCA analysis of the RNA sequencing data of three potato cultivars at 18 and 36 hpi with *A. solani*.

C – Control, T – A. solani Inoculated, MB – Magnum Bonum, D – Désirée, K – Kuras, 18 – 18h time point, 36 – 36h time point, 1, 2, 3 – Biological replicate

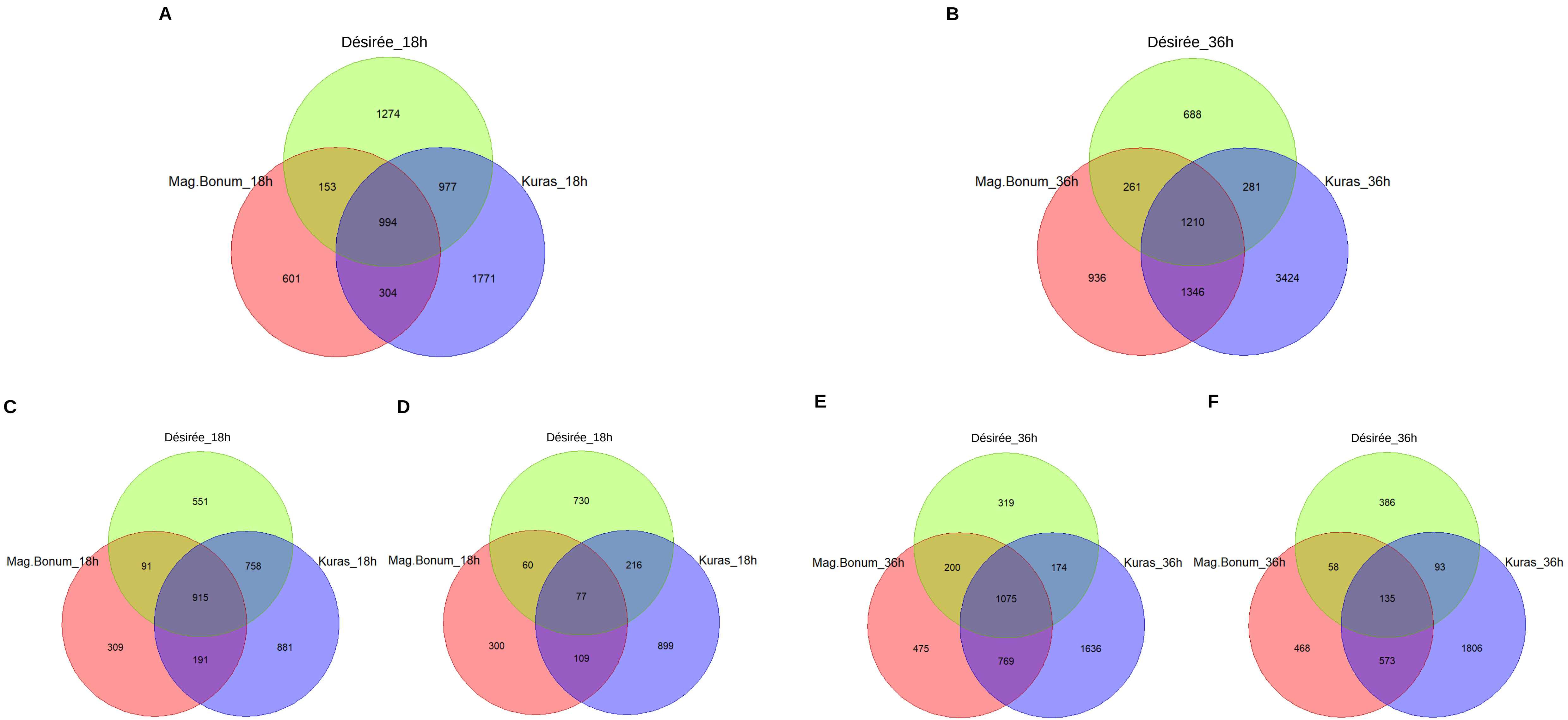

Figure S2: Venn diagrams showing the overlap between the DEGs of different potato cultivars at 18 and 36 hpi.

Mag.Bonum – Magnum Bonum; 18h and 36h – 18 and 36 hour post infection (hpi); (A&B) – Overlap of DEGs at 18 and 36 hpi, respectively, (C&D) – Overlap of up- and down- regulated DEGs at 18 hpi, (E&F) - Overlap of up- and down-regulated DEGs at 36 hpi.
